# Bioreactor cultivation of CHO DP-12 cells under sodium butyrate treatment – comparative transcriptome analysis with CHO cDNA microarrays

**DOI:** 10.1186/1753-6561-5-S8-P98

**Published:** 2011-11-22

**Authors:** Sandra Klausing, Oliver Krämer, Thomas Noll

**Affiliations:** 1 Institute of Cell Culture Technology, Bielefeld University, 33615 Bielefeld, Germany

## Background

Sodium butyrate (NaBu) is not only known to inhibit proliferation but also to increase the specific productivity in cultivation of Chinese hamster ovary (CHO) cells [1] – the most commonly used mammalian cell line for pharmaceutical protein production [2]. So far, little is known about the underlying mechanisms and genes that are affected by butyrate treatment. Besides the proteomic approach to unravel proteins involved in the processes, the analysis of transcriptomes presents another promising method. Here we show an application of our CHO cDNA microarray to identify genes associated with increased productivity during cultivation of CHO cells under sodium butyrate treatment.

## Materials and methods

Four batch cultivations of CHO DP-12 cells (clone # 1934, ATCC CRL-12445) were performed in 2 L bioreactor systems under pO_2_- and pH-controlled conditions. In the exponential growth phase, 67 hours after inoculation, 2 mM sodium butyrate was added to three processes. The fourth was left untreated to function as control culture. Samples were taken before and then repeatedly after the addition of butyrate. RNA was isolated from cell pellets of 5·10^6^ cells using TRIzol® Reagent (Invitrogen). For subsequent cDNA labeling, the Agilent Low-Input QuickAmp Labeling Kit (Agilent Technologies) was used. The custom designed 2 x 105 k cDNA microarray (Agilent Technologies) was spotted with 94,580 probes designed from CHO cDNA sequenced in-house. 38,310 of 41,039 sequenced contigs were used for the microarray, each covered by 2-4 probes [3]. Data analysis was done with ArrayLims, EMMA, and SAMS, three CeBiTec based software tools [4]. The raw data gathered by the microarray experiments were processed by standard Agilent background normalization and subsequent lowess normalization.

## Results

**Figure 1:**
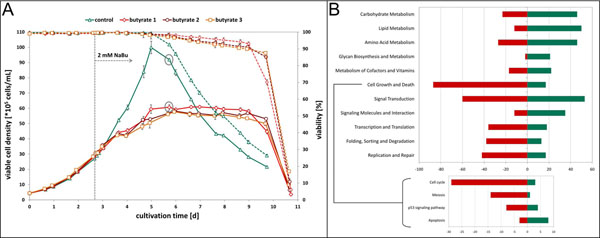
(**A)**: Concentration of viable cells and cell viabilities for the time course of CHO DP-12 batch processes. Error bars represent the standard deviation of triplicate measurements with the Cedex system (Roche Diagnostics). Red and orange lines represent biological replicates of cultures treated with 2 mM NaBu, the control process is shown in green. Dashed lines show viabilities. The grey arrow indicates the addition of NaBu, the grey circles show the sample points compared later in the microarray analysis (72 h of NaBu Treatment). (**B)**: Number of up- and downregulated genes of selected KEGG pathway categories with a detailed view of four pathways from the Cell Growth and Death KEGG category. Results show only those found as differentially expressed after filtering. Red: downregulated in NaBu cultures; green: upregulated in NaBu cultures (compared to control culture).

The control culture reached a maximum viable cell density of 1·10^7^ cells/mL while NaBu treated cells reached a plateau at about 6·10^6^ cells/mL and retained a viability above 90 % four days longer than untreated cells (Figure 1A). The three biological replicates of NaBu cultures yielded results with similar general trends. The maximum antibody concentration of the control culture was 110 mg/L whereas cells treated with NaBu reached a maximum of 175 mg/L antibody. 72 hours after addition of NaBu the specific antibody production rate was increased by a factor of 3.6 (NaBu culture: 4.5 pg/(cell·d)) compared to control culture (1.2 pg/(cell·d)).

Of this time point, samples were analyzed in microarray experiments. A significance test with FDR control (α=0.05) was carried out for the four technical replicates (including two dye-swaps) of the microarray. For analysis, the following filtering settings were chosen to identify differentially expressed genes: adjusted p-value ≤ 0.05, log-ratio < -1 or > 1 (equals fold change < -2 or > 2) and log-intensity ≥ 6 (equals ≥ 64 raw intensity). From a total of 1461 genes found to be differentially expressed under NaBu treatment, 771 genes were upregulated and 690 genes were downregulated (derived from EC numbers in KEGG pathways, Figure 1B). Many differentially expressed genes from pathways involved in carbohydrate, lipid, amino acid and glycan metabolism are upregulated which is most likely linked to higher productivity. A large portion of genes from pathways associated with cell growth and death are downregulated and most of these genes originate from cell cycle processes. This correlates with reports of cell cycle arrest under NaBu treatment [1]. Some examples of regulated genes are shown in Table 1.

**Table 1:** Fold change of selected genes from microarray analysis.

KEGG pathway category	Gene symbol	Description	Mean fold change in NaBu culture compared to control culture		# of probes
	CA150	Transcription factor CA150b	-3.31	↓	3
**Transcription & Translation**	Ccdc12	Coiled-coil domain containing 12	-2.01	↓	1
	Y14	RNA-binding protein 8A	2.14	↑	2

	Pkmyt1	Protein kinase, membrane associated tyrosine/threonine 1	-2.04	↓	2
**Cell Cycle**	c-Myc	Myc proto-oncogene protein	-3.44	↓	3
	Ink4c	Cdkn2c cyclin-dependent kinase inhibitor 2C (p18, inhibits CDK4)	3.04	↑	2
	CycD	Cyclin D1 (Ccnd1)	2.49	↑	1

	Pdcd4	Programmed cell death 4	3.89	↑	2
**Apoptosis**	Casp6	Caspase 6	3.06	↑	3
	PI3K	Phosphatidylinositol 3-kinase, regulatory subunit, polypeptide 1	2.44	↑	2

## Conclusions

Microarray analysis revealed a high number of regulated genes under sodium butyrate treatment in pathways like carbohydrate metabolism, cell cycle and signal transduction. Some of the regulated genes are promising targets for overexpression or knockdown/knockout experiments and we will further investigate the knockdown effect of selected genes using a siRNA approach in CHO cells. Our in-house microarray is suitable for further transcriptomic analysis of CHO cells under various conditions.
